# Clinical Insights into *RELA*-Associated Disease: From Genotype to Phenotype and Exploring Treatment

**DOI:** 10.3390/genes17091043

**Published:** 2026-08-29

**Authors:** Chun Pan, Cuifang Zheng, Yuhuan Wang, Jieru Shi, Lin Wang, Ying Huang

**Affiliations:** Department of Gastroenterology, Children’s Hospital of Fudan University, National Children’s Medical Center, No. 399 Wanyuan Road, Minhang District, Shanghai 201102, China; chunpan64@163.com (C.P.);

**Keywords:** *RELA*, inborn errors of immunity, hereditary autoinflammatory diseases, genotype–phenotype correlation

## Abstract

**Background/Objectives**: *RELA* encodes the p65 subunit of NF-κB and plays a critical role in immune regulation, epithelial protection, and anti-apoptotic signaling. Pathogenic *RELA* variants cause monogenic immune dysregulation with heterogeneous clinical manifestations. However, genotype–phenotype relationships and optimal treatment strategies remain incompletely defined. **Methods**: We conducted a comprehensive literature-based analysis of reported individuals with *RELA* variants and additionally described a family carrying a *RELA* c.706C>T (p.R236*) variant, including a clinically affected proband and his variant-positive father with isolated vitiligo. Clinical, immunological, genetic, endoscopic, and therapeutic data were extracted and summarized using a module-based phenotypic framework. Exploratory analyses were performed to examine potential genotype–phenotype patterns and reported treatment responses. **Results**: A total of 72 individuals with *RELA* variants, including the proband and his variant-positive father from the present family, were analyzed. *RELA*-associated disease exhibited marked clinical heterogeneity, encompassing mucocutaneous, systemic inflammatory, autoimmune, gastrointestinal, hematologic, allergic/eosinophilic, and infection-related manifestations. Exploratory analyses suggested that truncating or splice-site variants were more frequently observed among individuals with mucocutaneous lesions, whereas missense variants appeared to be more common among those with autoimmune manifestations. Tumor necrosis factor (TNF) inhibitors were among the therapies associated with favorable reported responses. In the present family, the proband presented with early-onset Behçet-like intestinal inflammation and achieved clinical and endoscopic remission after thalidomide and dose-escalated infliximab treatment. **Conclusions**: This study expands the clinical spectrum of *RELA*-associated disease and highlights preliminary variant-related clinical patterns that require confirmation in larger independent cohorts. The available treatment experience suggests that TNF blockade may be considered as a therapeutic option in selected patients, although comparative efficacy cannot be established from the available data.

## 1. Introduction

The Rel/NF-κB family is composed of five members: p65(RelA), RelB, c-Rel, p50/p105 (NF-κB1), and p52/p100 (NF-κB2) [1]. Its signaling pathway not only plays a crucial role in regulating immune and inflammation response, but also helps maintain intestinal mucosal homeostasis [2]. The *RELA* gene encodes p65/RelA, the key transcription factor of the classic NF-κB pathway, participates in the expression of proinflammatory cytokines, cell survival, and infection-related interferon signaling [3].

The *RELA* heterozygous variant was initially reported in a case of neonatal high bone mass [4]. Recent studies showed that *RELA* heterozygous variants can result in a class of monogenic diseases characterized by immune disorders. Patients with *RELA* variants often present with a clinical phenotype characterized by Behcet-like mucosal skin ulcers, as well as systemic lupus erythematosus (SLE)-like autoimmune manifestation. Patients may also have arthritis, recurrent infections, and other multisystem involvement [5]. As for the immune phenotypes, *RELA* variants are associated with enhanced Th1 response, elevated interferon (IFN) gamma levels and dysfunctions of Tregs cells [6,7].

*RELA*-associated disease is a rare monogenic immune dysregulation disorder, and its epidemiological characteristics remain poorly defined. Current knowledge is largely based on individual case reports and small familial cohorts, and reliable population-based estimates of disease prevalence and incidence are not currently available. Consequently, the clinical spectrum of *RELA*-associated disease remains incompletely defined, particularly with regard to genotype–phenotype correlations and therapeutic responses. The limited number of reported patients also makes it challenging to recognize clinical patterns and establish optimal treatment strategies. Here, we further performed a comprehensive literature review of previously reported patients with *RELA* variants combined with a newly identified family including one clinically affected proband and his variant-positive father, summarizing their genetic features, overlapping clinical manifestations, and documented treatment outcomes.

## 2. Materials and Methods

### 2.1. Patient Cohort

A child presenting with recurrent diarrhea was evaluated at the Children’s Hospital of Fudan University. Written informed consent was obtained from the patient’s legal guardians. This study was approved by the Ethics Committee of the Children’s Hospital of Fudan University.

### 2.2. Whole-Exome Sequencing (WES)

Peripheral blood samples from the proband and his parents were collected in EDTA-anticoagulated tubes, and genomic DNA was extracted for trio-based WES [8]. Exome capture was performed using the IDT xGen Exome Research Panel v2, followed by sequencing on an Illumina NovaSeq 6000 platform (Illumina, San Diego, CA, USA).

### 2.3. Structural Modeling

The structures of wildtype (WT) RELA protein and mutant RELA protein were modeled using AlphaFold 3 [9]. The mutant model was generated based on the predicted truncated RELA sequence. The predicted models were visualized and analyzed in PyMOL (3.1.0).

### 2.4. Literature Review

**Figure 1:**
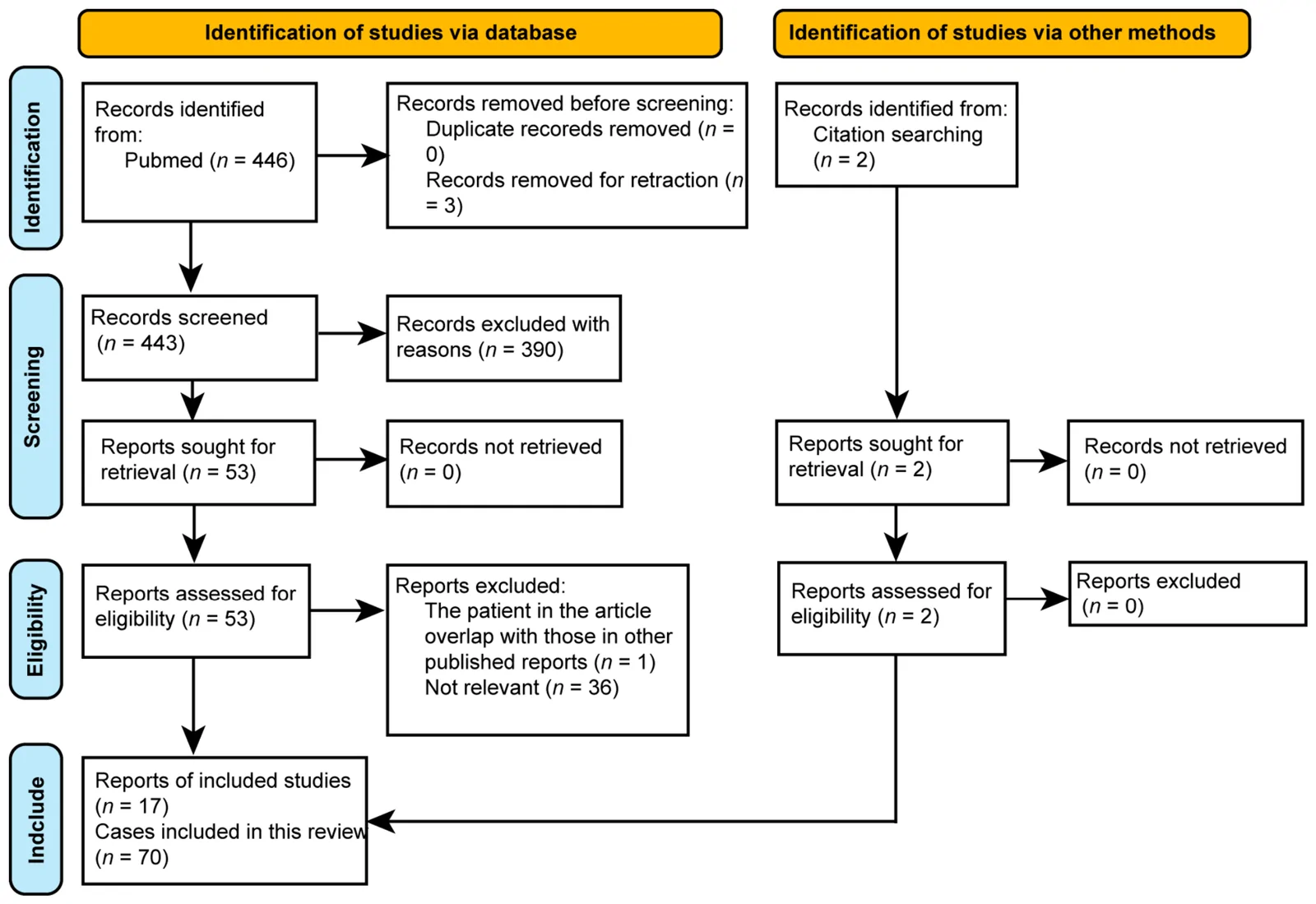
PRISMA flow diagram.

Following the Preferred Reporting Items for Systematic Reviews and Meta-Analyses (PRISMA) guidelines (Figure 1), a systematic literature search was performed in PubMed using the following search strategy: (RELA[Title/Abstract] OR “NF-kappaB p65”[Title/Abstract]) AND (mutation OR variant) AND (patient OR patients OR disease). The review protocol was not prospectively registered. Reference lists and citations of relevant publications were also screened to identify additional potentially eligible reports. Only publications written in English or Chinese were included. The final search was conducted on 22 February 2026.

After removal of duplicate records, titles and abstracts were screened for relevance, followed by full-text assessment of potentially eligible articles. Screening and data extraction were performed by the investigators according to predefined eligibility criteria, and ambiguous cases were discussed among the investigators to reach consensus. Studies were eligible if they reported human individuals carrying *RELA* variants with available clinical information. Articles were excluded if they involved only animal or in vitro models, lacked individual clinical data, were review articles without original cases, or were not available in full text. Conference abstracts were excluded because of insufficient clinical detail. Preprints were included only when they contained extractable case-level clinical and genetic data, fulfilled the same eligibility criteria as peer-reviewed publications, and did not substantially overlap with subsequently published reports. No formal methodological quality scoring was applied because the available evidence predominantly consisted of case reports and small-case series.

For patients reported in more than one publication, duplicated cases were identified on the basis of variant type, inheritance pattern, pedigree information, demographic features, and clinical descriptions, and were counted only once in the final analysis.

Treatment response was categorized according to the clinical outcomes explicitly reported in the original publications, as standardized response criteria were not consistently available. “Response” was assigned when complete or marked clinical improvement/remission was reported; “partial response” when improvement was reported but relevant disease manifestations persisted; and “no response” when no meaningful clinical improvement, persistent or progressive disease, or treatment discontinuation because of lack of efficacy was reported. “Used” indicates that the treatment was administered, but the available report did not provide sufficient information to determine the clinical response.

### 2.5. Phenotype Module Definitions

Given the marked clinical heterogeneity and overlap among reported *RELA*-associated phenotypes, clinical features were summarized using a descriptive, non-mutually exclusive module-based framework instead of a formal disease classification. The modules included mucocutaneous/skin lesions, systemic inflammation, gastrointestinal involvement, autoimmunity, hematologic/lymphoproliferative abnormalities, allergic/eosinophilic manifestations, and immunodeficiency or recurrent infections.

Mucocutaneous/skin manifestations included oral or genital ulcers and other reported skin lesions, whereas systemic inflammation included recurrent fever and elevated inflammatory markers such as erythrocyte sedimentation rate (ESR) or C-reactive protein (CRP). Gastrointestinal involvement was further categorized as intestinal Behçet’s disease/inflammatory bowel disease, nonspecific gastrointestinal manifestations such as diarrhea or abdominal pain without a specific gastrointestinal diagnosis, or other gastrointestinal disorders, including celiac disease and eosinophilic gastrointestinal disease. Autoimmune manifestations included clinically diagnosed autoimmune diseases, such as systemic lupus erythematosus and juvenile idiopathic arthritis, as well as clinical disorders or manifestations associated with elevated disease-related autoantibodies as reported in the original publications. Allergic/eosinophilic manifestations included peripheral blood eosinophilia, eosinophilic gastrointestinal disease, markedly elevated serum IgE levels, and allergic manifestations explicitly reported in the original publications. Immunodeficiency/recurrent infections included recurrent or chronic infections and deficiencies in serum immunoglobulins or immunoglobulin subclasses explicitly reported in the original publications. Hematologic/lymphoproliferative abnormalities included abnormalities in peripheral blood cell counts, including cytopenias, as well as lymphadenopathy, splenomegaly or hepatosplenomegaly, and clinically diagnosed lymphoproliferative disorders reported in the original publications.

Classification was based on clinical diagnoses and manifestations explicitly reported in the original publications, without re-interpretation of incomplete case descriptions or inference from unreported information. Individual patients could be assigned to more than one module when manifestations involved multiple clinical domains. Phenotype assignment was initially performed based on the available clinical descriptions, and ambiguous cases were reviewed and resolved by consensus. Formal inter-rater agreement was not assessed.

### 2.6. Statistics

Categorical variables are summarized as counts and percentages. Missing clinical data were not imputed. For phenotype frequency analyses, the total cohort size (*n* = 72) was used as the denominator, and unreported clinical features were considered unavailable rather than confirmed absent. For exploratory comparisons, *RELA* variants were further grouped into a truncating/splice-site group and missense group. Comparisons of categorical variables across variant classes were performed using Fisher’s exact test because of sparse cell counts. Odds ratios and 95% confidence intervals were calculated for selected phenotype modules. P values were adjusted using the Benjamini–Hochberg method. All analyses were considered exploratory. All statistical analyses were performed using R software (4.6.0).

## 3. Results

### 3.1. Clinical Characteristics of the Family Reported in This Study

The family included the proband (P1) and his father (P2), both of whom carried a *RELA* variant. P1 was a 1-year-old Chinese male born to non-consanguineous parents. He initially presented with fever and infectious diarrhea. Laboratory evaluation showed persistent systemic inflammation and elevated serum immunoglobulins (Supplementary Table S1). Endoscopic evaluation revealed a giant ulcer in the ileocecal region (Supplementary Figure S1A). Throughout the disease course, recurrent cutaneous abscesses required surgical intervention, as well as eczematous rashes involving wrist and ankle regions were reported. Thalidomide provided partial clinical improvement (Supplementary Figure S1B), whereas dose-escalated infliximab (IFX) achieved clinical remission and mucosal healing (Supplementary Figure S1C). P2, the father of P1, had a history of vitiligo but no other reported symptoms. Detailed information on the clinical course is provided in Supplementary Data and Supplementary Figure S1.

### 3.2. Identification and Interpretation of the RELA Variant

WES was performed in the proband and his parents to investigate the genetic basis of the disease. A heterozygous nonsense variant in *RELA*, c.706C>T (p.R236*), was identified in the proband and inherited from his father. This variant is extremely rare in the Genome Aggregation Database (gnomAD v4.1.0), with an allele frequency of 1.86 × 10^−6^. Based on the American College of Medical Genetics and Genomics (ACMG) guidelines, the variant was classified as pathogenic (PVS1+PM2+PP4). According to ClinVar, the variant has been classified as pathogenic. In silico prediction also supported a potentially deleterious effect, with a CADD score of 37 and a MutationTaster score of 1. In addition, AlphaFold 3-based structural modeling was performed to illustrate the predicted structural consequences of the premature truncation (Supplementary Figure S1D). Further functional studies are required to determine the biological significance of these predicted structural changes.

### 3.3. Demographic and Genetic Characteristics of Reported Patients

The clinical features of patients with *RELA* variants reported to date were systematically reviewed. A total of 17 studies comprising 70 patients were included. Together with the 2 individuals identified in the present family, 72 patients were analyzed [4,5,6,7,10,11,12,13,14,15,16,17,18,19,20,21]. Overall, 35 patients were female and 37 were male, indicating a relatively balanced sex distribution among patients with *RELA* variants (Supplementary Table S2). Regarding age at onset, nine patients had adult-onset disease, nine had unknown onset age, and the remaining 54 patients presented during childhood. Among them, 34 (63%, 34/54) had disease onset before 6 years of age.

**Figure 2:**
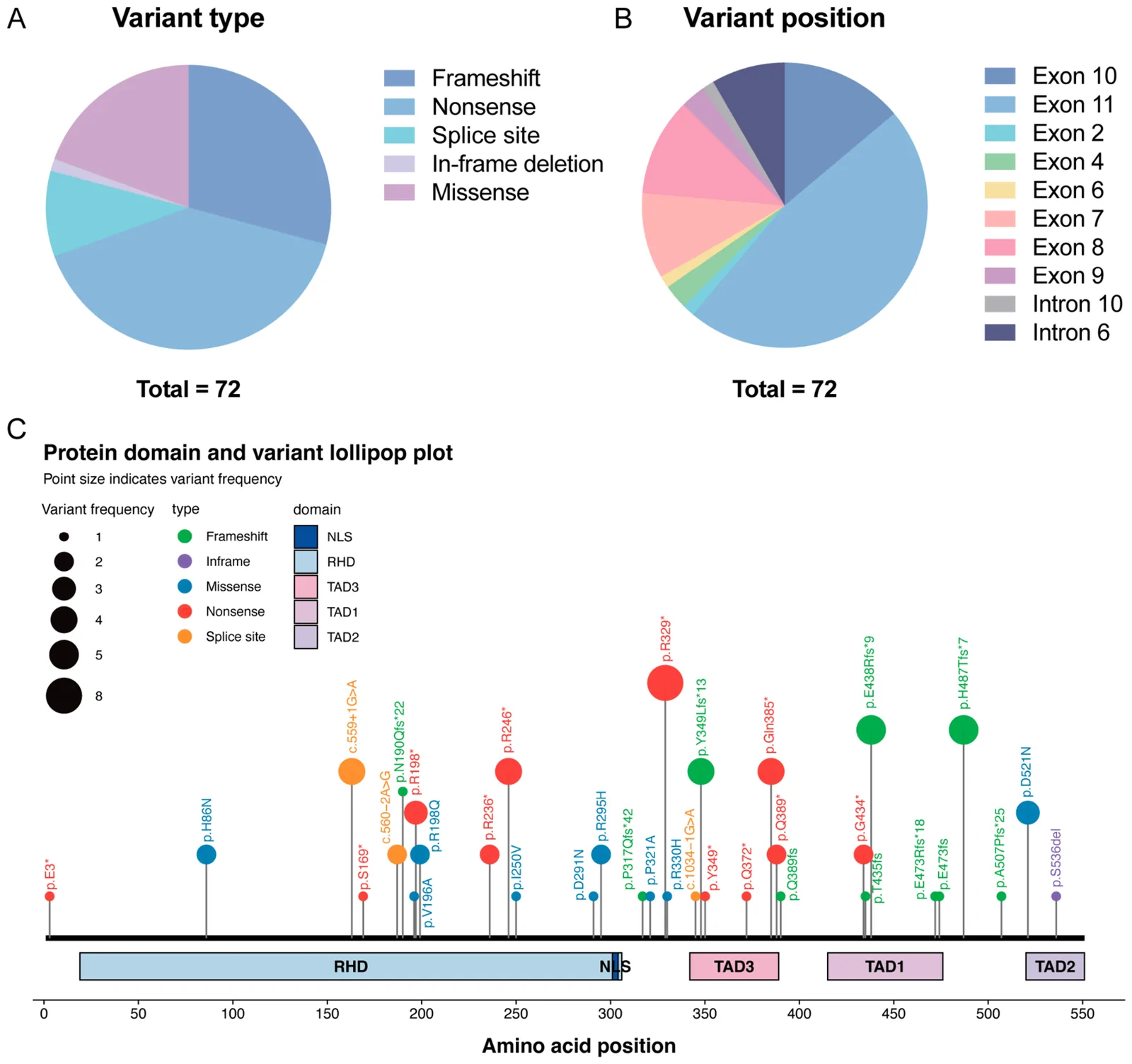
Spectrum of *RELA* variants in reported patients. (**A**) Variant types in reported patients. (**B**) Variant positions in reported patients. (**C**) Lollipop plot of reported *RELA* variants.

Most patients carried truncating variants (57/72), including nonsense variants (29/72), frameshift variants (21/72), and splice-site variants (7/72). Missense variants were found in 14 patients, and one patient carried an in-frame deletion (Figure 2A). At the nucleotide level, variants clustered mainly within exon 11 (Figure 2B). At the protein level, reported variants were distributed across the Rel homology domain (RHD) and C-terminal transactivation domain (TAD)-containing region (Figure 2C).

### 3.4. Clinical Manifestations Across the RELA-Associated Disease Spectrum

**Figure 3:**
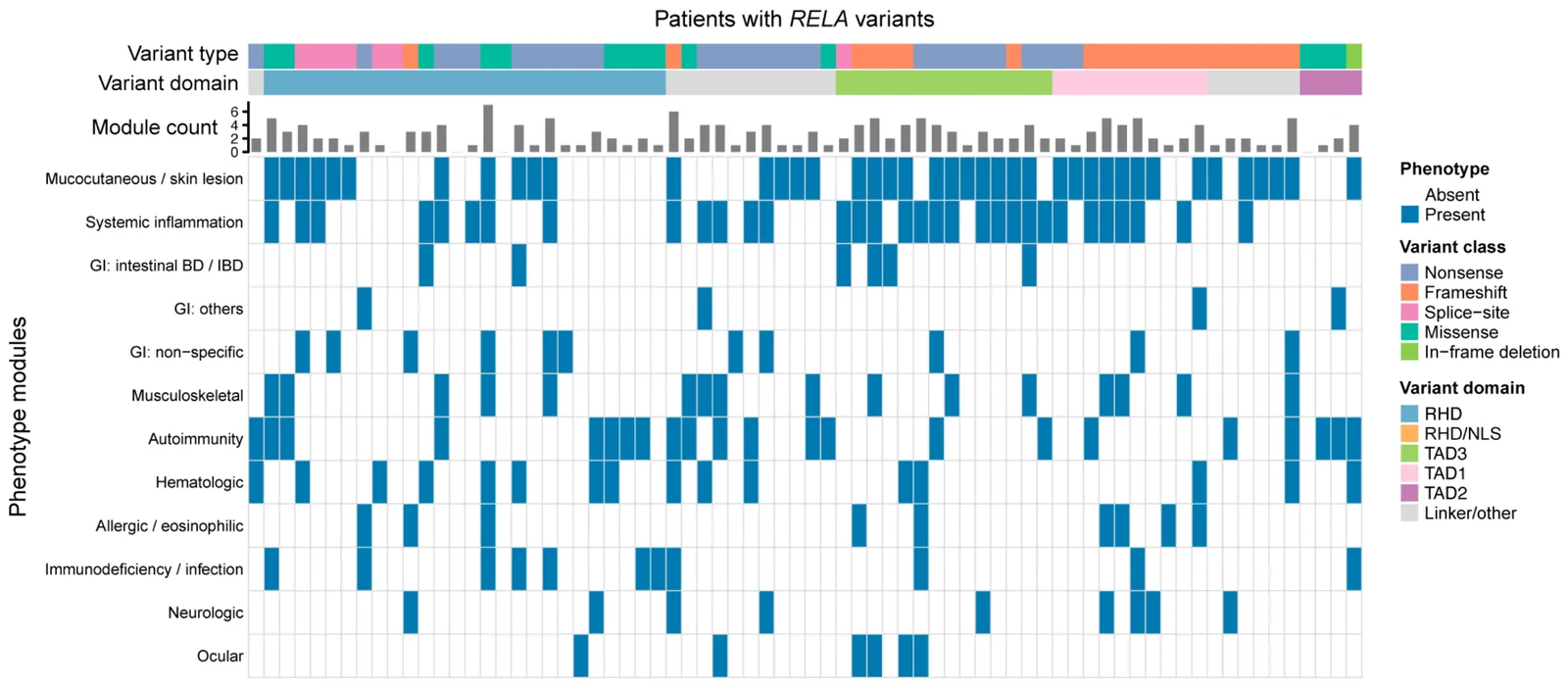
Phenotype modules of patients with *RELA* variants.

Clinical manifestations were highly heterogeneous and frequently overlapped across patients. Therefore, we summarized the reported phenotypes using a non-mutually exclusive module-based framework (Supplementary Table S3 and Figure 3). Mucocutaneous or skin lesions represented the most frequently reported module, occurring in 41 patients (56.9%). Systemic inflammatory manifestations, including recurrent fever or autoinflammatory features, were reported in 32 individuals (44.4%). Gastrointestinal (GI) involvement was observed in 21 patients (29.2%). Among these, 11 patients had non-specific gastrointestinal symptoms, whereas six patients had intestinal ulcers or were diagnosed with inflammatory bowel disease, as well as single cases of eosinophilic gastrointestinal disease and coeliac disease. Autoimmune or autoantibody-associated manifestations, including SLE/SLE-like disease and other clinically relevant autoimmune features, were reported in 22 patients (30.6%). Hematologic and lymphoproliferative abnormalities were also common and were observed in 16 patients (22.2%). Allergic-like or eosinophilic manifestations were identified in nine patients (12.5%), while immunodeficiency or recurrent infection was reported in 11 patients (15.3%).

### 3.5. Exploratory Genotype–Phenotype and Age-Related Patterns

**Figure 4:**
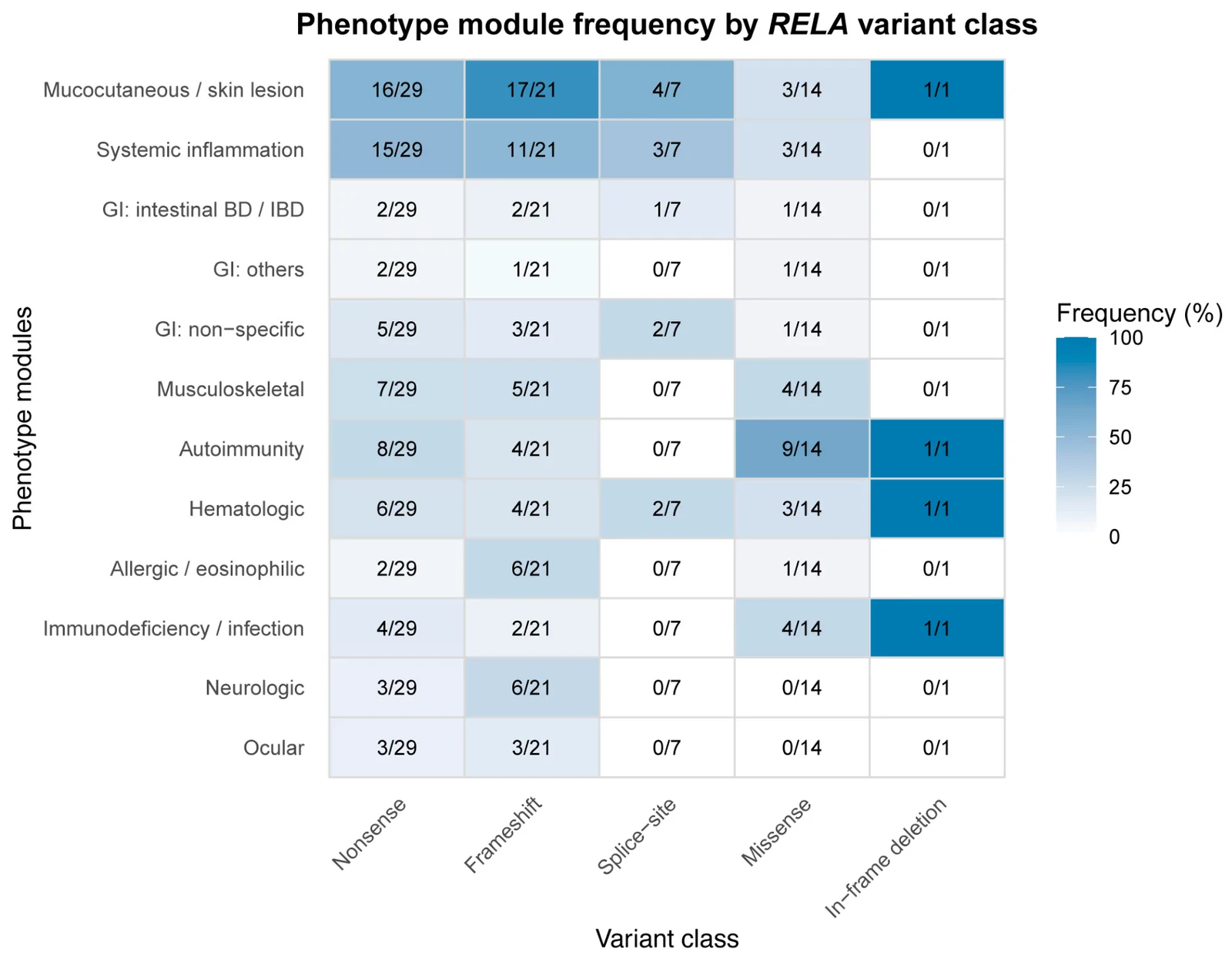
Heatmap of phenotype module frequency by *RELA*-variant types. Values shown in each cell represent the number of affected individuals divided by the total number of individuals in the corresponding variant subgroup (n/N). Color intensity indicates the corresponding phenotype frequency (%).

In an exploratory genotype–phenotype analysis, mucocutaneous lesions were more frequently reported in patients with truncating or splice-site variants than in those with missense variants (37/57,64.9% vs. 3/14, 21.4%; Fisher’s exact test, *p* = 0.006, FDR = 0.028). This difference remained statistically significant after FDR correction. Systemic inflammatory features were also more frequently observed in the truncating or splice-site group at the nominal significance level (29/57, 50.9% vs. 3/14, 21.4%; *p* = 0.007), but the difference did not remain significant after FDR correction (FDR = 0.238). By contrast, autoimmune manifestations were more common among patients carrying missense variants than among those with truncating or splice-site variants (9/14, 64.3% vs. 12/57, 21.1%; *p* = 0.003, FDR = 0.028) (Figure 4, and Supplementary Table S4). In contrast, GI involvement did not show an obvious variant-type preference.

**Figure 5:**
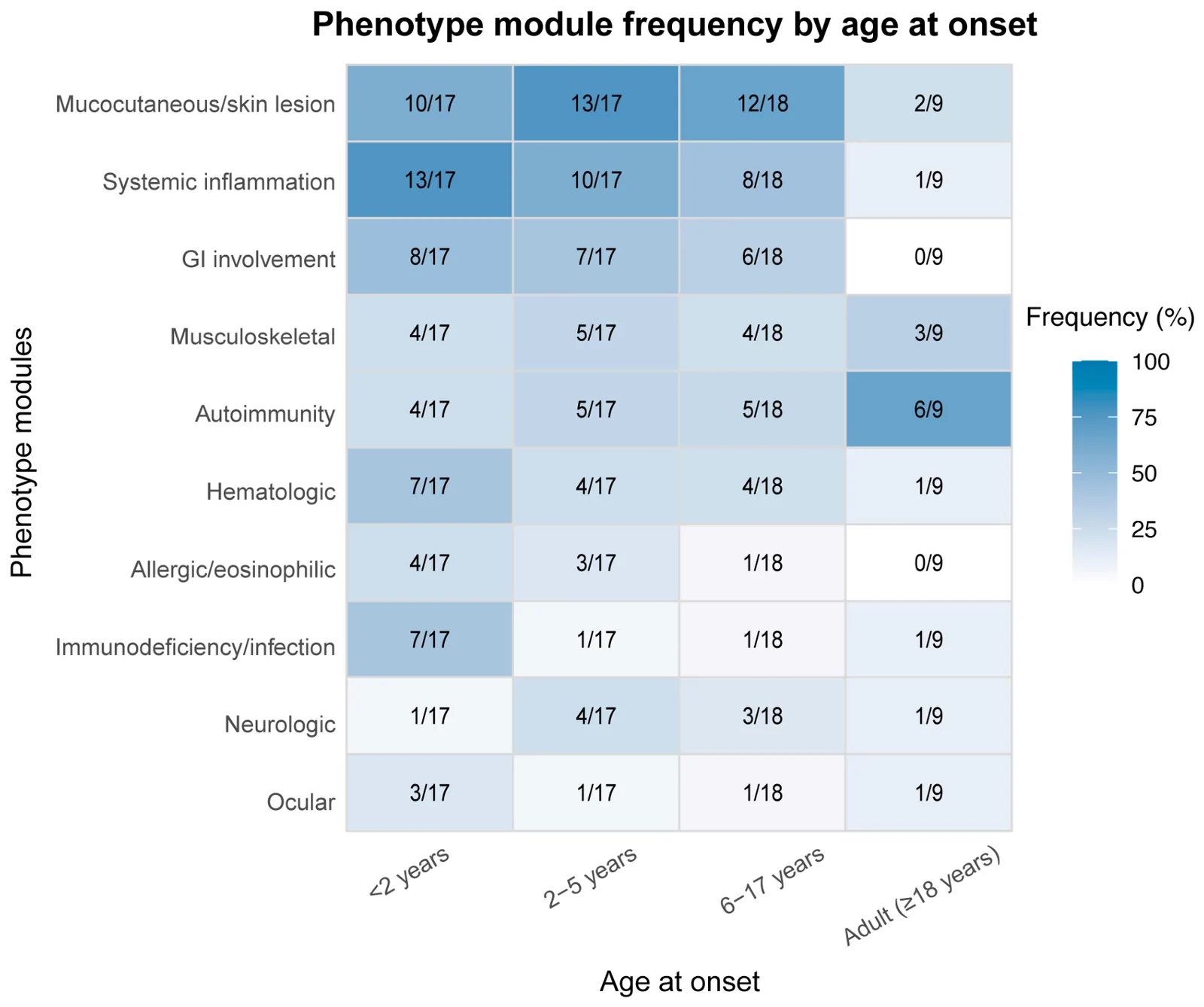
Heatmap of phenotype module frequency according to age at disease onset. Values shown in each cell represent the number of affected individuals divided by the total number of individuals in the corresponding age subgroup (n/N). Color intensity indicates the corresponding phenotype frequency (%).

Age-stratified analysis further suggested that systemic inflammation, GI involvement, hematologic abnormalities, and immunodeficiency or infection-related features were more frequently reported in patients with disease onset before 2 years of age, whereas autoimmune manifestations appeared to be relatively more frequent among adult-onset cases (Figure 5, and Supplementary Table S5). Detailed numerical data for all phenotype comparisons are provided in Supplementary Tables S4 and S5. These findings should be interpreted as exploratory rather than definitive genotype–phenotype or age-related associations.

### 3.6. Treatment Strategies and Clinical Responses

Treatment responses were heterogeneous across reported patients (Table 1 and Supplementary Table S6). Glucocorticoids were the most frequently reported therapy and were used in 28 patients. Clinical improvement was described in several cases, whereas relapse during dose reduction, steroid dependence, or incomplete response was also reported. Conventional immunomodulatory or anti-inflammatory agents, including thalidomide and hydroxychloroquine, were used in a subset of patients and showed variable responses.

Biologic or targeted therapies were reported in fewer patients. TNF inhibitors were the most commonly reported biologic agents (*n* = 19), and clinical improvement was described in several treated patients (*n* = 16). Other targeted therapies, including IL-1 blockade (*n* = 3), IL-6 blockade (*n* = 3), JAK inhibitors (*n* = 3), IL-4/IL-13 blockade (*n* = 1), and IL-5 blockade (*n* = 1), were each used in only a small number of patients, with variable reported outcomes. Overall, treatment responses differed substantially among reported patients.

## 4. Discussion

We report a family carrying a *RELA* nonsense variant. Previous studies have suggested that *RELA* deficiency impairs NF-κB signaling, thereby weakening mucosal protection and anti-apoptotic signaling and predisposing patients to recurrent oral/genital ulcers, cutaneous lesions, fever, and gastrointestinal inflammation [5]. *RELA* encodes the p65 subunit of the canonical NF-κB pathway, which contains a Rel homology domain (RHD) responsible for DNA binding, dimerization, nuclear localization, and interaction with IκB proteins, as well as a C-terminal transactivation domain (TAD) required for transcriptional activation [22]. Therefore, different *RELA* variants may affect NF-κB activity through distinct mechanisms depending on their location and functional consequences. However, because patient-derived samples from the proband were unavailable, functional validation of the *RELA* p.R236* variant could not be performed. Therefore, the proposed pathogenic mechanism remains hypothetical and requires further experimental validation. In the proband, disease onset occurred at 1 year of age, with early manifestations dominated by large ileocecal ulcers and recurrent fever episodes, followed by recurrent oral ulcers. Thalidomide was associated with marked improvement in oral ulcers, while IFX contributed to control the intestinal ulcers. Taken together, these features are consistent with previously reported mucocutaneous and the gastrointestinal spectrum of *RELA*-associated disease and further illustrate the clinical presentation of early-onset Behçet-like inflammation. In addition, the isolated vitiligo observed in the father carrying the same *RELA* variant is also within the previously reported phenotypic spectrum [12], highlighting the variable expressivity of *RELA*-associated disease. More broadly, an underlying monogenic disorder, including *RELA*-associated disease, may be considered in children with early-onset Behçet-like manifestations, particularly in the presence of atypical clinical or immunological features.

Our literature review provides preliminary observations on possible genotype–phenotype and age-related clinical differences in the clinical presentation of *RELA*-related diseases. Truncating or splice-site variants appeared to be more frequently associated with mucocutaneous lesions and systemic inflammatory manifestations, whereas autoimmunity seemed to be relatively enriched among patients carrying missense variants. These observations may reflect differences in the biological effects of different *RELA* variants. Truncating and splice-site variants may affect RELA functional dosage through mechanisms such as nonsense-mediated decay, potentially leading to haploinsufficiency, although the precise functional consequences may vary depending on the individual variant [5]. In cases where mutant proteins escape degradation and remain stable, additional mechanisms, including dominant-negative effects or altered protein interactions, cannot be excluded. Conversely, missense variants may produce more selective functional alterations depending on the affected residue and domain. The p.R236* variant identified in the present family introduces a premature termination codon within the RHD. If the mutant transcript escapes nonsense-mediated decay, the predicted truncated protein would lack the downstream region of the RHD and the entire TAD, potentially affecting DNA binding, dimerization, nuclear regulation, and transcriptional activation. However, the actual molecular consequence of this variant remains to be determined experimentally. In exploratory age-stratified analyses, patients with disease onset before 2 years of age more often showed systemic inflammation, gastrointestinal involvement, hematologic abnormalities, and immunodeficiency or recurrent infection-related features, suggesting that very-early-onset *RELA*-associated disease may represent a more severe multisystem immune dysregulation phenotype. These observations raise the possibility that age at onset may help stratify the clinical presentation of *RELA*-associated disease. The limited number of reported patients, incomplete phenotypic descriptions, and the small sizes of the missense and adult-onset subgroups preclude firm genotype–phenotype conclusions. Very-early-onset cases may require earlier genetic evaluation and more comprehensive immunological assessment, particularly when systemic inflammation, gastrointestinal disease, hematologic abnormalities, or recurrent infections coexist. In contrast, adult-onset cases appeared to be relatively enriched for autoimmune manifestations. The marked phenotypic variability among individuals carrying *RELA* variants likely reflects the complexity of NF-κB regulation. Differences in residual RELA activity, variant-specific effects on individual functional domains, genetic modifiers, epigenetic regulation, environmental triggers, and tissue-specific requirements for NF-κB signaling may all influence disease expression. Cell-type-specific effects of RELA dysfunction may contribute to phenotypic variability, with impaired epithelial and stromal cell survival potentially favoring mucosal disease and immune-cell dysregulation contributing to systemic autoinflammatory or autoimmune manifestations [5,7,23]. The limited number of reported patients, incomplete phenotypic descriptions, potential family clustering, and the small number of missense and adult-onset cases preclude firm genotype–phenotype conclusions. Thus, these findings should be considered exploratory and require validation in larger cohorts with standardized clinical and functional characterization.

From a therapeutic perspective, the management of *RELA*-associated disease should be interpreted in light of its underlying biology. *RELA* deficiency may impair NF-κB-mediated epithelial protection and anti-apoptotic signaling, thereby increasing mucosal susceptibility to TNF-driven inflammatory injury [5,23]. Beyond epithelial injury, dysregulated NF-κB signaling may also disturb immune tolerance by altering lymphocyte activation, survival, and inflammatory cytokine production [24]. Together, these mechanisms may contribute to autoimmune manifestations and provide a plausible rationale for TNF blockade. In our literature review, clinical improvement was reported in 16 of 19 treatment episodes involving anti-TNF agents. However, these observations were derived from heterogeneous case reports and should not be interpreted as evidence of superiority over other therapeutic approaches. In the proband (P1), infliximab treatment resulted in sustained clinical remission and achieved endoscopic remission with mucosal healing in our patient.

Glucocorticoids were the most frequently used agents, with 28 treatment episodes recorded, and often provided short-term symptomatic control. However, relapse during tapering, steroid dependence, and the need for steroid-sparing therapy limited their long-term utility. Colchicine was also commonly used, with six responses and seven non-responses, suggesting variable efficacy and possible benefit in selected patients. In contrast, conventional immunomodulators showed inconsistent efficacy. Azathioprine appeared to have limited benefit, whereas methotrexate and thalidomide were effective only in some individual cases. In our case, thalidomide induced a partial clinical response. Evidence for targeted therapies other than TNF blockade remains limited. These agents were used only in a small number of patients, often based on the dominant clinical phenotype, and the reported outcomes were variable. Therefore, their efficacy in *RELA*-associated disease remains unclear and cannot be reliably assessed from the currently available cases.

These findings suggest that *RELA*-associated disease is unlikely to be uniformly driven by a single downstream cytokine pathway. Instead, treatment should be individualized according to the dominant clinical phenotype, with TNF blockade currently representing one of the most consistently reported treatment approaches associated with clinical improvement.

Several limitations should be acknowledged. First, this study was based on a retrospective literature-based analysis of published cases and is therefore subject to publication bias, reporting bias, and incomplete case-level information. The literature search was limited to PubMed, and additional databases were not searched, which may have resulted in the omission of potentially relevant reports. Mildly affected or asymptomatic *RELA* variant carriers may have been underreported, whereas patients with severe or unusual phenotypes may have been preferentially published. Second, clinical data were extracted from reports with variable diagnostic work-up, follow-up duration, laboratory evaluation, clinical characterization, diagnostic criteria, and treatment assessment. In some disease-specific cohort studies, *RELA* variants were identified without comprehensive multisystem phenotyping, such that unreported manifestations may reflect incomplete assessment rather than true absence. This heterogeneity in phenotypic ascertainment, together with incomplete reporting of clinical features, may have resulted in underestimation or misclassification of specific clinical features and influenced the reported phenotype frequencies. In addition, the phenotype module framework used in this study was developed for descriptive synthesis of heterogeneous clinical manifestations rather than as a formally validated classification system. Phenotype assignment was based on reported clinical information, and formal inter-rater agreement was not assessed, which may have introduced classification variability. Third, several reported individuals belonged to the same families, and the observations were therefore not fully independent; family clustering, shared genetic background, and environmental modifiers may have influenced the apparent genotype–phenotype associations. Fourth, the exploratory subgroup analyses were limited by small sample sizes, particularly for missense variants, adult-onset cases, and individual treatment categories. Finally, treatment responses were derived from heterogeneous case reports without standardized response criteria, dosing regimens, treatment duration, follow-up periods, or objective outcome measures, and many patients received combination therapies. Therefore, the reported responses to TNF blockade cannot establish comparative efficacy and may have been influenced by publication, reporting, and indication bias.

Future studies should prioritize the establishment of prospective multicenter registries with standardized clinical, immunological, genetic, and treatment-related phenotyping. Such collaborative efforts would improve the completeness and consistency of case-level data and provide a more reliable basis for investigating potential genotype–phenotype relationships. Variant-specific functional studies are also needed to determine how different *RELA* variants affect protein expression, NF-κB signaling activity, transcriptional regulation, and cellular responses. In parallel, standardized definitions of treatment response, objective outcome measures, and longer-term follow-up will be important for comparing therapeutic strategies and informing individualized management of *RELA*-associated disease.

## 5. Conclusions

In summary, our literature-based analysis expands the recognized clinical spectrum of *RELA*-associated disease and provides a descriptive overview of possible variant-related clinical differences, although these observations should be considered exploratory and hypothesis-generating and require validation in larger, independent, well-characterized cohorts.

## Figures and Tables

**Table 1:** Treatment summary of reported *RELA* variants.

Medication	Treated Patients, n/N (%)	Response not Reported, n/N (%)	Partial Response, n/N (%)	Response, n/N (%)	Non-Response, n/N (%)
GC	28/72 (38.9%)	11/28 (39.3%)	1/28 (3.6%)	14/28 (50.0%)	2/28 (7.1%)
anti-TNF	19/72 (26.4%)	1/19 (5.3%)	2/19 (10.5%)	14/19 (73.7%)	2/19 (10.5%)
COL	18/72 (25.0%)	5/18 (27.8%)	0/18 (0.0%)	6/18 (33.3%)	7/18 (38.9%)
THAL	4/72 (5.6%)	1/4 (25.0%)	1/4 (25.0%)	2/4 (50.0%)	0/4 (0.0%)
AZA	7/72 (9.7%)	0/7 (0.0%)	0/7 (0.0%)	1/7 (14.3%)	6/7 (85.7%)
MTX	9/72 (12.5%)	5/9 (55.6%)	1/9 (11.1%)	2/9 (22.2%)	1/9 (11.1%)
MMF	6/72 (8.3%)	1/6 (16.7%)	0/5 (0.0%)	4/5 (80.0%)	1/5 (20.0%)
RTX	5/72 (6.9%)	1/5 (20.0%)	0/5 (0.0%)	3/5 (60.0%)	1/5 (20.0%)
IVIG	3/72 (4.2%)	0/3 (0.0%)	0/3 (0.0%)	3/3 (100.0%)	0/3 (0.0%)
HCQ	4/72 (5.6%)	2/4 (50.0%)	0/4 (0.0%)	1/4 (25.0%)	1/4 (25.0%)
IL-1 blocker	3/72 (4.2%)	0/3 (0.0%)	0/3 (0.0%)	1/3 (33.3%)	2/3 (66.7%)
IL-6 blocker	3/72 (4.2%)	1/3 (33.3%)	0/3 (0.0%)	0/3 (0.0%)	2/3 (66.7%)
JAKi	2/72 (2.8%)	0/2 (0.0%)	1/2 (50.0%)	0/2 (0.0%)	1/2 (50.0%)
HSCT	1/72 (1.4%)	0/1 (0.0%)	0/1 (0.0%)	1/1 (100.0%)	0/1 (0.0%)

Note: GC, glucocorticoids; COL, colchicine; THAL, thalidomide; AZA, azathioprine; MTX, methotrexate; MMF, mycophenolate mofetil; RTX, rituximab; IVIG, intravenous immunoglobulin; HCQ, hydroxychloroquine; JAKi, janus kinase inhibitors; HSCT, hematopoietic stem cell transplantation.

## Data Availability

All data generated or analyzed during this study are included in this published article and its Supplementary Files.

## Supplementary Materials

The following supporting information can be downloaded at: https://www.mdpi.com/article/10.3390/genes17091043/s1, Supplementary Data: The detailed clinical course of P1; Figure S1: Clinical features and structural impact of a novel *RELA* variant. (A) Representative images of gastrointestinal endoscopy before treatment. (B) Representative images of colonoscopy after thalidomide. (C) Representative images of colonoscopy after six IFX treatments. (D) Computer modeling of WT RELA protein and Mutant RELA protein; Table S1: The immune phenotype of P1; Table S2: The demographic and genetic characteristics of reported patients; Table S3: Detailed clinical features of reported cases; Table S4: Genotype–phenotype comparison between truncating/splice-site and missense *RELA* variants; Table S5: Age-stratified comparison of phenotype module frequencies among individuals with *RELA* variants; Table S6: Detailed treatments and responses of reported cases.

## Abbreviations

The following abbreviations are used in this manuscript:

TNF	Tumor necrosis factor
SLE	Systemic lupus erythematosus
IFN	Interferon
WES	Whole exome sequencing
WT	Wild type
IFX	Infliximab
ACMG	American College of Medical Genetics and Genomics
RHD	Rel homology domain
TAD	Transactivation domain
GI	Gastrointestinal
FDR	False discovery rate
Vs.	Versus
