# Supplementary material for: Clinical Insights into RELA-Associated Disease: From Genotype to Phenotype and Exploring Treatment
Source: Genes (Basel). 2026 Aug 29;17(9):1043. doi: 10.3390/genes17091043 (PMC13606629; doi:10.3390/genes17091043)
Supplement: Supplementary file 1 [file genes-17-01043-s001.zip › Supplementary file S1.pdf]

**Supplementary Data**

**The detailed clinical course of P1**

P1. The proband presented with fever and infectious diarrhea at age of 1 year. After anti-infective treatment, recurrent fever persisted, and he was subsequently referred to our center for further evaluation. Laboratory investigations showed persistent systemic inflammation, including elevated C-reactive protein (CRP, 21–50 mg/L), increased erythrocyte sedimentation rate (ESR, 66 mm/h). Fecal calprotectin was also elevated (392 μg/g). Echocardiography and bone marrow aspiration revealed no significant abnormalities, and tuberculosis was excluded. Immunological evaluation revealed elevated serum IgG, IgA, and IgE levels, together with increased circulating pro-inflammatory cytokines, including TNF-α, suggesting a state of immune activation and dysregulation. Detailed immunological findings are summarized in Supplementary Table 1. Endoscopic evaluation revealed extensive gastrointestinal (GI) involvement, characterized by ulcerations involving approximately 3/4 of the ileocecal region and about half of the terminal ileum (Supplementary Figure 1A).

Based on the combination of recurrent fever and severe ileocecal ulceration, an autoinflammatory disease was suspected. Prednisone was administered and effectively controlled the recurrent fever. Exclusive enteral nutrition (EEN) combined with mesalazine was initiated to manage the intestinal inflammation. Thalidomide was subsequently introduced at a dose of 1.6 mg/kg to maintain inflammatory control during steroid tapering. During this period, the patient had no recurrent fever or diarrhea; however, at two years of age, recurrent oral ulcers developed, and routine blood testing revealed neutropenia. However, during thalidomide therapy, the patient developed fatigue. And after 6 months of treatment, a follow-up endoscopy revealed partial remission of intestinal ulcers (Supplementary Figure 1B). Considering the partial endoscopic response and thalidomide-related adverse effects, the dose of thalidomide was subsequently reduced to 0.75mg/kg. Treatment was therefore escalated to infliximab (IFX) at an induction dose of approximately 5mg/kg. Therapeutic drug monitoring before the fourth infusion showed a concentration of 2.5 ug/mL with no anti-drug

antibodies. Consequently, the IFX dose was increased to approximately 8mg/kg every 8 weeks. The patient underwent a repeat endoscopy showing mucosal healing before the sixth IFX infusion (Supplementary Figure 1C), indicating a favourable response to IFX therapy.

During the disease course, recurrent cutaneous abscesses required surgical intervention, as well as eczematous rashes involving wrist and ankle regions were reported. During a recent follow-up, his parents discontinued thalidomide because of his hand tremors occurring during sleep, after which oral ulcers recurred.

**Supplementary Figure**

**Supplementary Figure S1.** Clinical features and structural impact of a novel *RELA* variant. (A) Representative images of gastrointestinal endoscopy before treatment. (B) Representative images of colonoscopy after thalidomide. (C) Representative images of colonoscopy after 6 times IFX treatments. (D) Computer modeling of WT RELA protein and Mutant RELA protein.

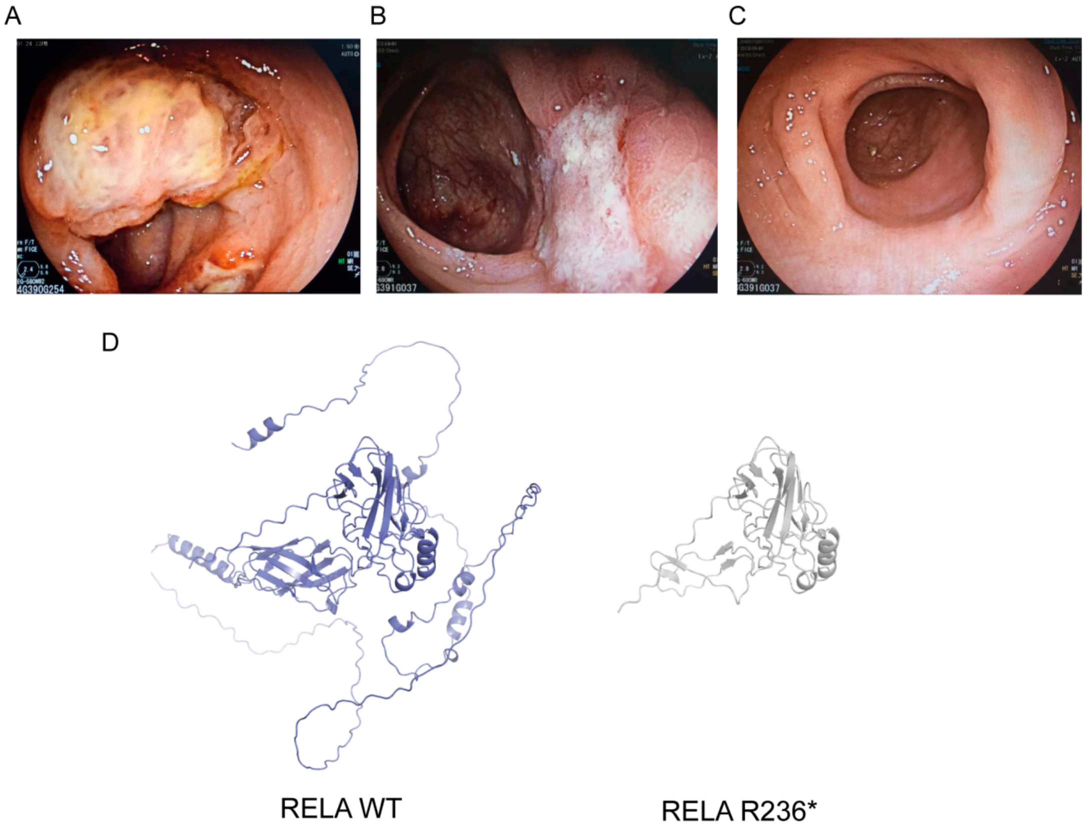
A
B
C
D
RELA WT
RELA R236*
